# The contribution of Physician Assistants in primary care: a systematic review

**DOI:** 10.1186/1472-6963-13-223

**Published:** 2013-06-18

**Authors:** Mary Halter, Vari Drennan, Kaushik Chattopadhyay, Wilfred Carneiro, Jennifer Yiallouros, Simon de Lusignan, Heather Gage, Jonathan Gabe, Robert Grant

**Affiliations:** 1 Faculty of Health and Social Care Sciences, Kingston University and St George’s, University of London, Cranmer Terrace, London SW17 0RE, UK; 2 Department of Primary Care & Public Health Sciences, King’s College London, 7th Floor, Capital House, 42 Weston Street, London SE1 3QD, UK; 3 Department of Health Care Management and Policy, University of Surrey, Guildford GU2 7XH, UK; 4 School of Economics, University of Surrey, Guildford GU2 7XH, UK; 5 Centre for Criminology and Sociology, Royal Holloway University of London, Egham, Surrey TW20 0EX, UK

**Keywords:** Physician assistants, Family practice, Physicians, Family, General practice, Primary health care, Review

## Abstract

**Background:**

Primary care provision is important in the delivery of health care but many countries face primary care workforce challenges. Increasing demand, enlarged workloads, and current and anticipated physician shortages in many countries have led to the introduction of mid-level professionals, such as Physician Assistants (PAs). Objective: This systematic review aimed to appraise the evidence of the contribution of PAs within primary care, defined for this study as general practice, relevant to the UK or similar systems.

**Methods:**

Medline, CINAHL, PsycINFO, BNI, SSCI and SCOPUS databases were searched from 1950 to 2010. Eligibility criteria: PAs with a recognised PA qualification, general practice/family medicine included and the findings relevant to it presented separately and an English language journal publication. Two reviewers independently identified relevant publications, assessed quality using Critical Appraisal Skills Programme tools and extracted findings. Findings were classified and synthesised narratively as factors related to structure, process or outcome of care.

**Results:**

2167 publications were identified, of which 49 met our inclusion criteria, with 46 from the United States of America (USA). Structure: approximately half of PAs are reported to work in primary care in the USA with good support and a willingness to employ amongst doctors. Process: the majority of PAs’ workload is the management of patients with acute presentations. PAs tend to see younger patients and a different caseload to doctors, and require supervision. Studies of costs provide mixed results. Outcomes: acceptability to patients and potential patients is consistently found to be high, and studies of appropriateness report positively. Overall the evidence was appraised as of weak to moderate quality, with little comparative data presented and little change in research questions over time.

Limitations: identification of a broad range of studies examining ‘contribution’ made meta analysis or meta synthesis untenable.

**Conclusions:**

The research evidence of the contribution of PAs to primary care was mixed and limited. However, the continued growth in employment of PAs in American primary care suggests that this professional group is judged to be of value by increasing numbers of employers. Further specific studies are needed to fill in the gaps in our knowledge about the effectiveness of PAs’ contribution to the international primary care workforce.

## Background

Primary care is important to the delivery of health care [1,2]. The major domains of clinical primary care are first contact care and entry into the health care system [1], continuous and ongoing patient focused care for a defined population, coordination of care and comprehensiveness of services [3]. Clinical primary care is increasingly being required to deliver both on public health policies of health promotion and prevention and also chronic disease management strategies [1]. The combination of increasing patient and policy demands, current and approaching medical shortages as well as access issues in remote and rural areas has placed the primary care workforce in sharp focus in many countries [1,4]. While in some systems clinical primary care is only provided by medical practitioners, known as general practitioners or family doctors [5], others have developed team approaches of differently skilled types of staff some including mid-level practitioners such as nurse practitioners and medical assistants [3]. In some settings, such as rural areas with shortages of doctors, the mid-level practitioners become the first contact providers of primary care [6,7]. One such group of mid-level practitioners is physician assistants (PAs).

PAs were introduced in the United States of America (USA) in the 1960s in response to medical shortages and misdistribution [8] and over 70,000 are now employed in health services [9]. PAs are health professionals, with a PA qualification, who undertake physical examinations, investigations, diagnosis and treatment within their scope of practice as agreed with their supervising doctor [8]. In the USA PAs also have prescribing rights [8]. Since the 1970s demand for PAs has outstripped supply, particularly from solo primary care practices [10]. A recent survey by the American Academy of Physician Assistants (AAPA) of 19,830 PAs (less than a quarter of their membership) found that 2,966 were employed in family medicine and another 1,768 in family medicine with urgent care provision, with 25% employed in solo practice physician offices and rural and community health centres [11]. Over the last decade other countries have started to see small numbers of PAs in their health care workforce and have been exploring the contribution PAs could make in their health care system [12]. The employment of PAs in general practice/family medicine has been reported in Canada [13], Netherlands [14], Australia [15] and the UK [16], albeit in small numbers and in isolated developments [17].

The introduction of new professional groups into the health care workforce raises questions for the public and for health service managers and planners as to their deployment, effectiveness, safety and cost. One challenge in considering evidence from a range of health care systems is the applicability to country-specific health care delivery systems. Previous reviews of evidence concerning physician assistants [18-25] were of limited applicability to the UK general practice setting. The objective of this systematic review was to appraise the published evidence as to the contribution (numbers, retention, employability, consultation type, activity levels, impact on the work of others, cost, acceptability to patients, appropriateness of care) of PAs within primary care, defined for this study as general or family practice, in comparison to physicians or nurses if data are available, drawing on studies of any method.

## Method

This review of evidence was undertaken in accordance with the guidance for reviews of health care produced by the Centre for Reviews and Dissemination [26]. Seven English language electronic databases were searched (from 1950 or their start date if later) Medline (1950), CINAHL (1981), Embase (1996), PsycINFO (1987), BNI (2004), SSCI (1955) and Scopus (2004) to the date of this review’s last search (14/9/10), using search terms as detailed in Table 1.

**Table 1:** Search terms for each database (as described or entered into each database)

**Principle for the search**	**Database**	**Index terms**	**Additional keywords**
Physician Assistant	MEDLINE	Physician Assistants	physician* assistant*
	EMBASE	physician assistant	physician* assistant*
	CINAHL	Physician Assistants	physician* assistant*
		Physician Assistant Attitudes	
		American Academy of Physician Assistants	
	PsycINFO	-	physician* assistant*
	BNI	**-**	physician* assistant*
	SSCI	**-**	physician* assistant*
	SCOPUS	**-**	physician* assistant*
Primary Care	MEDLINE	Primary Health Care	general practi*
		Physicians, Family	
		Family Practice	
	EMBASE	primary medical care	primary care
		general practice	family practice
			general practi*
	CINAHL	Primary Health Care	general practi*
		Physicians, Family	
		Family Practice	
	PsycINFO	Primary Health Care	primary care
		Family Physicians	family practice
	BNI	-	primary care
			general practi*
			family practice
	SSCI	-	primary care
		-	primary health care
			primary medical care
			general practi*
			family practi*
	SCOPUS		primary care
			primary health care
			primary medical care
			general practi*
			family practi*
English language	All	Limiters: English Language	

The asterisk * was used to truncate the search term. For example, physician* assistant* will search for physician assistant, physician’s assistant, physician assistants, etc.

We used the definition of general practice/family medicine provided by the European Region of the World Organisation of Family Doctors (WONCA Europe) [27] though we used a broad range of key terms within our initial literature search (Table 1). An example of a full search (from Medline) is presented in Table 2.

**Table 2:** Search carried out in MEDLINE

**Search ID#**	**Search terms**	**Search options**
S9	S6 and S7	Limiters- English Language
		Search modes – Boolean/Phrase
S8	S6 and S7	Search modes – Boolean/Phrase
S7	S3 or S4	Search modes – Boolean/Phrase
S6	S1 or S2 or S5	Search modes – Boolean/Phrase
S5	(MH “Family Practice”)	Search modes – Boolean/Phrase
S4	Physician* assistant*	Search modes – Boolean/Phrase
S3	(MH “Physician Assistants”)	Search modes – Boolean/Phrase
S2	General practi*	Search modes – Boolean/Phrase
S1	(MH “Primary Health Care”) OR (MH “Physicians, Family”)	Search modes – Boolean/Phrase

The asterisk * was used to truncate the search term. For example, physician* assistant* will search for physician assistant, physician’s assistant, physician assistants, etc.

Electronic search results were downloaded into bibliographic software, screened by at least one of three researchers (MH, KC, VMD) with at least 10% receiving a quality check, using the predefined inclusion and exclusion criteria. Inclusion criteria were: that the PA role was based on the medical model with a recognised PA qualification, the setting included general practice/family medicine (including community paediatrics in the USA), the findings relevant to ‘family medicine’ were presented separately from findings meeting a broader definition of ‘primary care’; and the publication was a journal article. Exclusion criteria were that: the setting was secondary care, in-patient care, outpatient care or ambulatory care (that is, consultations, treatments, tests etc. in the USA that are similar to UK outpatient activity); the activities were primary care specialty activities (USA) that would be regarded as secondary care in the UK, or according to the WONCA definition [26] (that is care provided by Obstetricians/Gynaecologists, Internists or Primary Care Physicians); the PAs were still in training; the personnel were nurses or others who had trained as ‘medical assistants’ for a particular disease condition without a recognised PA course. We also excluded papers that did not distinguish between PAs and other providers (such as physicians and nurse practitioners) in its presentation of results or the paper did not distinguish the data from the general practice/family medicine setting within the presentation of results from ‘primary care’ overall; and the publication was a report or thesis.

Relevant citations were retrieved in full, where available. Two researchers independently assessed each retrieved paper (MH, KC, WC, JY) with a third researcher mediating where eligibility was uncertain or there was disagreement (VMD, MH), and other members of the research team offered expert advice on health systems where this was required to enable the researchers to make decisions (HG, SdeL).

A data extraction framework (variables for which data were sought) was developed based on describing the study in terms of its aim, setting, PAs’ activities, method/s, population, sample size and key findings regarding the contribution (including interventions and outcomes) of PAs to primary care, and was piloted on five studies (MH, KC, WC). The framework proposed by Donabedian (1988) for assessing the quality of care was used as a basis for investigating evidence of the contribution of PAs. This distinguishes factors related to the structure (personnel and their qualifications, facilities and equipment, fiscal and operational policies); process (elements of consultations, technical competency, roles and responsibilities, coordination and continuity and acceptability to those receiving the care); and outcomes (patient health and wellbeing, survival, rehabilitation, recovery, satisfaction, perceived appropriateness and cost effectiveness) of care [28].

Due to the wide variety of types of studies retrieved, a broad quality assessment of the studies was undertaken using the Critical Appraisal Skills Programme (CASP) tools [29] with the additional questions from the British Medical Journal guidance for peer reviews: “Do the interpretation and conclusion follow from the findings?” and “Do you believe the results?” [30] Ratings of evidence were made qualitatively, from a judgment of the combination of answers to the CASP and additional questions. A study would be described as strong if each CASP question could be answered fully, medium if the answer was yes to each question based on scant reporting or weak if answering the CASP questions highlighted gaps e.g. in method/description of method or results.

As very few studies reported on the same interventions or outcomes, no summary measures could be made. Assessment of bias is included in the description of major strengths and limitations in Additional files 1, 2 and 3.

**Figure 1:**
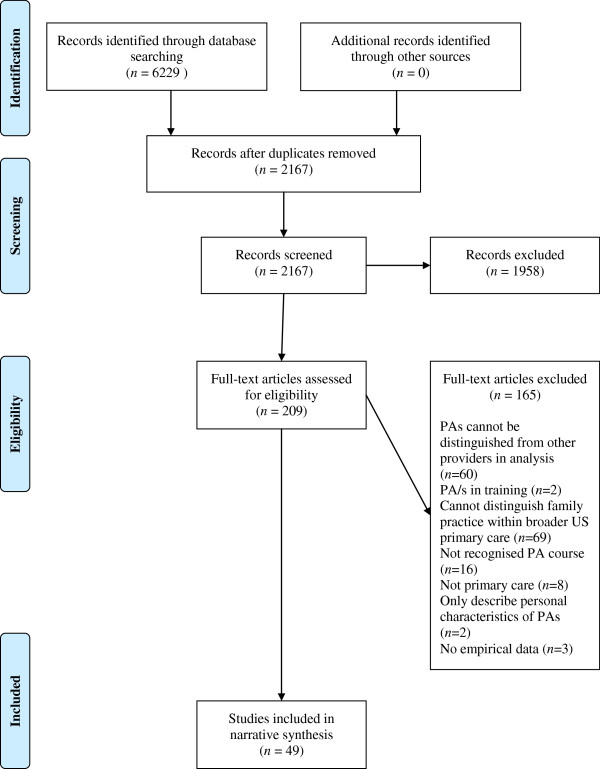
PRISMA flowchart.

Studies were not excluded on the basis of their quality assessment. The review report conforms to the Preferred Reporting Items for Systematic Reviews and Meta-Analyses (PRISMA) [31]. A PRISMA flowchart [31] detailing the numbers of studies at each stage of the search was used (Figure 1).

## Results

Forty nine studies were included in the review, 46 of which are from the USA and one each from the UK, the Netherlands and Australia, as presented in the PRISMA [31] flowchart (Figure 1). Twenty seven report from quantitative surveys (five of which are secondary analyses), five from medical record reviews, three from structured/quantitative observations of practice, four from interview or focus groups (three of which are qualitative), and one from administrative cost data only. The remaining nine are mixed method studies. The heterogeneity in study type, populations and outcome measures precluded any meta-analysis and a narrative review was undertaken. The narrative account of synthesised findings is organised into evidence related to the structure, process and outcome of PAs in primary care. The term ‘doctor’ is used to cover the range of terms – physician, family physician, family practitioner and general practitioner – used to describe the medical practitioners in the studies we included.

Additional files 1, 2 and 3 provide detail for each study – study design, study aim, setting, population, sample (size), key findings (referring to interventions and outcomes) and major strengths and limitations.

### Evidence concerning questions of the structure of care involving PAs in primary care

The measures of structure reported in the studies focused predominantly on PAs as a human resource in primary care (Additional file 1), investigating numbers, retention and willingness to employ, and physician and managers’ views on the value of or barriers to employing PAs.

#### Numbers of PAs working in primary care

The studies that report on the numbers of PAs employed in primary care (*n* = 11), range in publication date from 1977 to 2007, are all from the USA (Additional file 1) and inform about the growing numbers of PAs and uncertainty about the proportion working in primary care. Three studies report about PA employment in a single state, graduates of a single university and a single health maintenance organisation [32-34]. These studies report that between 49% and 61% of primary care PAs work in family medicine, general or family practice according to our definition. Nine studies report on national surveys or secondary data analyses conducted by professional organisations [35-43]. Chronologically the sample number surveyed or in routine datasets increased with each study. Surveys of PA graduates in 1977 and 1978 reported 44% [35] and 52% [36] working respectively in family practice. The numbers of PAs forming the population for later studies is significantly higher, but these studies invited only a sample of the population to participate [37] or do not report the response rate for the data they conduct secondary analyses upon [38,39,41,42]. Four of these studies used the American Association of Physician Assistant (AAPA) databases and annual census and report that between 30–40% of PAs are practising in family medicine [37-39,41]. A later study reports 73% then 56% at different points in time [43]. Only two of these studies provide any comparative data with doctors or other mid level providers, one of which reports double the proportion of PAs to Nurse Practitioners (NPs) in a small sample in family medicine [38], while the other reports a very similar proportion of NPs as PAs in family practice in a large dataset [41]. A survey of randomly selected family physicians reports 33% of its respondents to be working with PAs [42].

#### Retention of PAs in general practice

The evidence about retention is limited to two small studies from the USA [44,45]. One observational study reports that 14 of 17 PAs had been working in the same practice for one year or more [44] and a qualitative interview study suggests that confidence in their ability to provide adequate health care, a desire for small town life, and residing in and being involved in the community influence PAs to remain in primary care in remote locations [45].

#### Clinician support and willingness to employ PAs

The level of support from doctors for the PA role, the reasons for this, and willingness to employ PAs is reported in eight American studies. Support for the concept of the role is reported as high amongst family practice doctors [46]. The reasons stated for employing a PA are numerous, with the following appearing in more than one study: a) size of the patient caseload [47,48] and the potential to decrease the doctor’s own workload [49], b) improvements to care [47-50] with reduced waiting times [50], c) potential to increase the number of patients cared for or other measures of practice productivity [47,48,50], d) increased doctor time for complex tasks [47,48,50] and e) increased patient satisfaction [50], with the role being liked and well accepted by patients [51]. Another study reports favourable views from 30 PA employers of both the need for PAs in rural and urban areas and also the quality of training and the usefulness of PA services [47]. One small observational study suggests some NPs view PA employment less favourably [51] although PAs themselves considered NPs to be less qualified for a mid level role [52].

Two studies give contradictory findings about the willingness of doctors who do not currently employ a PA. In the USA 61% of final year family practice residents (over half of whom were already familiar with the PA role) were reported as willing to hire a PA [49]. However, just 20% of ‘office based doctor’ respondents are reported as willing to employ a PA, with slightly more supporting a conditional trial of a PA in their practice on a low salary, in a contemporary study [10]. One UK study [53] reports in part on staff interviews and, similarly to the majority of the studies from the USA, suggests that doctors, nurses and non-clinical staff appreciated PAs’ enthusiasm and input to the practices. The limited description of both methods and findings regarding these interviews in this paper restricts any more detailed comparison with the other literature.

#### Structural barriers to the employment of PAs

The perceived structural impediments to PA practice are reported in five studies from the USA [46,47,49,50,54]. The reasons include: a poorer doctor-patient relationship or lower quality care [46,49], increased administration [47], malpractice claim fears [47,49,50], third party payment limitations [54], federal regulations [54], and patient [50] or medical community [54] opposition.

#### Summary of evidence regarding the structure of care

The apparent growth over time in the USA suggests that PAs have been increasingly chosen by doctors and health service managers to be employed in family medicine. However the quality and comprehensiveness of the samples and method improvements over time may also impact on these findings. The studies that explore doctors’ willingness and intention to employ PAs in family medicine in the USA and the UK suggest that support for the role is relatively high in some groups – particularly amongst those already employing a PA or with experience of working with PAs. Some ambivalence is also reported and a need for more evidence about the role in practice.

### Evidence concerning questions of the process of care involving PAs in primary care

The measures of process in care the studies report are mainly in the classification of the activities of PAs (*n* = 18), the activity level or patient throughput of the PAs (*n* = 8), the support or supervision required for PAs (*n* = 5) and the PAs’ impact on the workload of others (*n* = 4). Other process measures reported are PAs’ use of other healthcare services for their patients (*n* = 1) and the cost of a PA in a service (*n* = 2) (Additional file 2).

#### Patient consultation types

The largest group of papers (*n* = 18) offers descriptive accounts of the type of work activities performed by PAs [44,48,51,53,55-68]; all except two [53,68] are from USA, and the majority provide no comparative data with physicians or other staff the PAs work with (Additional file 2). These studies use survey, observation and medical record review methods with small and large samples and provide widely varying levels of detail about the clinical activities, age groups and other clinical and non-clinical activities undertaken by PAs. When the patient presenting conditions are classified as acute, chronic or preventative, the majority of studies report on acute conditions [44,55,56,58-60,63,65], ranging from conditions of unknown severity on presentation [59] to minor conditions [44,55,58-60,63,65]. Preventative and well person or insurance checks are also reported to form a large proportion of the PA workload [44,51,61,62,64-66], while providing care to patients with chronic conditions is reported less frequently amongst these USA studies [56,59,61]. Other studies make more general statements about the PAs’ type of activity [48,60] or provide long lists by condition type rather than severity [64,66].

The first PA graduates in the 1970s are reported to spend a large proportion of their patient contact time with paediatric patients [51,61,63] and young adults [63].

The activities of PAs vary by the size of the population served, with those in smaller communities carrying out a wider range of activities [66].

Comparative data with other groups present a picture of different consultation types between PAs and doctors: PAs see patients of all levels of complexity but patients are selected for consultations with PAs by presenting complaint [61]; doctors working in the same general practices as PAs attend more chronic, fewer acute conditions [58,67] and more serious problems [44], and PAs see patients with a younger age profile than that of those seen by doctors [67]. Similarity of the distributions of acute, chronic and preventative conditions seen by the PA with those seen by a nurse is suggested [56]. Doctors are reported to see more patients of higher socio economic status and white ethnicity than PAs [57].

Non clinical or indirect clinical activities are also reported in a small number of studies and include paperwork [51], documentation relating to the patient visit and consulting the doctor [58], administrative and data collection [61], and patient education, dispensing medication and specialist referrals [66].

The two non-USA studies present a similar picture in terms of providing detailed lists of condition groupings of patients seen by PAs [68], and, like the USA studies, lack any description of the severity of condition [53,68]. Both the non-USA studies state that the PAs attend more patients with unspecified conditions [53,68] than doctors, but similarity with the role of the nurse is disputed [53]. The younger patient age profile is also replicated in the Netherlands [68].

#### Activity level of PAs in primary care

The studies (*n* = 10) presenting data on the activity level are mostly evaluations of local schemes and use various methods and unit measures of throughput for PAs (Additional file 2), making synthesis of the results difficult. Contextual factors about the practice are not utilised within papers’ analyses, also limiting comparisons across studies.

In summary, the descriptive data without comparators suggest that a range of average numbers of patients is consulted per day by the PA (19 to 30 reporting from one of three decades each) [51,63,66]. Those studies that use within-practice comparisons report that PAs have a patient load comparable with other (unspecified) clinic staff [60], or a slightly higher patient consultation rate than nurse practitioners [38,41]. It is also suggested that the numbers seen per week increase by one third from one year to the next with the introduction of the PA in the family medicine practice [56]. Finally, regarding patient throughput, modelling of the reasons for the variability concludes that the reason for the visit, the number of tasks performed in the consultation, patient age and payment source are predictive of time spent [69]. Comparison with the studies from outside of the USA is limited by different units of measurement used. A UK study suggests that PAs achieve close to equivalence in individual capacity of a doctor in general practice in the UK [53], while 60% of the throughput of a GP in whole time equivalence is reported in the Netherlands [68].

#### PAs’ impact on the workload of others

The impact of the PA on the workload of others has been considered in two ways: the productivity and caseload of the employing physician and the support or supervision required for the PA role (*n* = 8) (Additional file 2).

Three studies published in the 1970s consider physician productivity, two of which report positive impact on the workload of others in modelling of the potential productivity of a physician comparing PA and non-PA employing practices [55] and in a before and after study [58]. However, the physician’s time on supervisory matters is also seen to have increased with the introduction of PAs [58] and 80% of PAs’ patient consultations also involved a nurse [61].

Outside of the USA and at least two decades later, a UK study suggests that eight of the nine general practices in the study had an increase in overall practice list size (number of patients registered) ranging from 2.4 to 5.3% in the one year following employment of the PA/s, with the PAs carrying out an average of 16.5 consultations per day against the GPs’ average of 17 [53]. Further, PAs are taking on tasks previously performed by GPs, although this is not quantified [53]. This redistribution of the physician’s tasks is examined in more detail in the Netherlands (in a report of the activity of a single, USA trained PA in one practice) with GPs observed to see greater numbers of older patients with undifferentiated diagnoses after introduction of the PA [68].

Support and/or supervision of the PA is reported on in seven studies, which show similar results for different PA configurations with the use of either clinical (medical record) data or observation methods, though all of these studies are from the late 1970s or early 1980s. All studies from the USA report a low rate of immediate support or supervision required in patient care episodes. The highest reported was 20% of patient care episodes either seen by or discussed with the physician by PAs in the first six months of practice with an unspecified sample [70]. In studies using observation, 12% of PA consultations are described as seen or discussed with the physician [69] for immediate consultation for ‘selected diagnoses’ [59] or for the 28 most common complaints as observed [61]; and for all workload analysed from clinical records [60,62]. Two studies seek to describe this supervision contact – the first [61] suggests that for patient sequences involving a PA, the physician tasks were usually taking a partial history, performing a partial examination or writing a prescription. The one study from the UK (where USA-trained PAs were working in UK general practice without prescribing rights) is more recent and describes 36-68% of all contacts from PA to GP about a particular patient as seeking a signature on a prepared prescription, and 1-16% of cases consulted as a review of treatment plans [53].

#### Use of other healthcare services and cost

One study attempts to measure the impact of PAs on the primary care system through their use of other healthcare services. Using patient encounter data and patient health survey data from six practices in one USA County at points in a three year period, PAs are reported to increase the tendency to hospitalise insured versus uninsured patients [56]. The sample size is not explicit in this paper, and the tendency is not quantified.

The issue of cost associated with employing a PA and the impact of the PA on practice finances is addressed in papers (*n* = 8) mainly from the 1970s and early 1980s and one from the 2000s. A number of studies suggest that PAs are expensive to employ or reduce profits. One study notes that, while the average total cost per patient episode was not related to the type of provider, PAs accrue significantly higher medication and laboratory costs than other providers, and this was most noticeable in patients with poor outcomes of care [71]. Four studies report low revenue per patient encounter [60,62,67,72], although the reasons for this or its interpretation differ, for example undercharging [60] or the PA undertaking tasks that are time consuming yet simple and therefore less remunerative [71]. Three descriptive studies suggest that in most cases the PA contributes positively to practice revenue/profit and quantify that small profit, comparing direct costs and overheads against patient visit revenue [59], also taking the patient throughput [63] and same-task ratio (PA: physician) [67] into account.

#### Summary of evidence regarding the process of care

The deployment of PAs has largely been to address the acute patient workload usually undertaken by doctors in family practice in the USA and in early development in the UK and the Netherlands. The extent to which these are undifferentiated conditions of unknown severity or minor and self limiting conditions is sometimes determined by the setting and the primary care practice’s operational policies regarding triaging patients with differently presenting problems to doctors, PAs or others. The evidence on productivity is mixed, regardless of country of origin, with some authors suggesting lower productivity by PAs compared to physicians, some suggesting similar rates of consultation and others stating that PAs increase the capacity or productivity of a practice. These studies are mainly descriptive and do not control for any factors found likely to influence throughput, limiting reliance on the absolute figures they provide. Studies which considered efficiency, examined through the impact of employing PAs on the workload and activities of the doctors in a practice, show that physician productivity may increase and indeed change focus with the introduction of a PA. However, this may be countered by the evidence that PAs work to a supervising doctor where supervision or advice is requested by the PA for up to one in six patients. The time spent in supervision of PAs by doctors was reported to be highest in recently qualified PAs or USA-trained PAs working in the UK (related to the absence of prescribing rights) and least for those with more experience working in their home country. In a more localised sense evidence is presented about the economic aspect of PAs in family practice. The evidence is mixed and USA-specific and challenging to transfer to other systems for funding family practice.

### Evidence concerning questions of the outcomes of care involving PAs in primary care

The measures of outcome reported in the studies were in two main groups: the acceptability of PAs to patients (*n* = 10) and the appropriateness of their care (*n* = 6). Details of the studies are given in Additional file 3.

#### Acceptability of PAs to patients

Ten studies investigate or include measures of the acceptability of the PA role, either in hypothetical situations, or of the PA’s care where the patient had seen a PA (Additional file 3). The evidence presented in these studies is relatively consistent in that the PA was acceptable to the majority of respondents/participants studied across the four decades of PA research.

Where the patient had been treated by a PA the level of satisfaction with the encounter was reported to be very high in a small interview study [45], high in medium sized survey studies [60,65,72,73] and in one large study of Medicare recipients [74,75] with very similar results for NPs and physicians. The evidence is more mixed in one focus group study of community residents in an area where the PA had been the sole primary care provider for the previous two years, with the residents suggesting that they would sometimes prefer to see a doctor due to: a) not having confidence in the PA (not being a doctor), b) already having a doctor or c) having a long term condition requiring specialist care [45].

Studies from the USA in which acceptability of the PA role was posed as a hypothetical question in interviews of general householders report positive findings [76,77]. These were tempered a little by decreasing willingness to see a PA over a physician for more complex conditions or those having greater severity [77]. An Australian study reports that 99% of patients presented with choice of provider versus time delays to consultation scenarios state that they would elect to see a PA, even when the scenario time delay to seeing a doctor was reduced [78]. The difference to the USA studies is that this Australian sample was naïve to the concept of the PA role [78].

#### Appropriateness of care provided by PAs

The evidence about the appropriateness of care provided by PAs is weak addressing this measure in several studies (*n* = 7, Additional file 3), all from the USA. Five of the studies report positive outcomes for PAs [44,48,59,72,73] with all bar one from the 1970s, although two of these are purely statements from either other health care professionals that patients were ‘adequately and appropriately treated’ [73] or from interviews with family practice faculty and residents who generally thought PAs provide high quality care [48]. The other three studies, again from the 1970s, provide quantitative comparisons of the PAs’ care against NPs and/or physicians of different training levels and report equivalence of care: no significant difference in the control of hypertension by PAs and physicians in a chart review study [59], highly correlated diagnostic and therapeutic appropriateness scores from observation of PAs and their employing physicians [44] and no significant difference in self reported patient functional status outcomes across PAs, family practice residents or family practice faculty physicians [72].

The positive findings are however not universal, with poor documentation of history and physical examination reported at a remote clinic in the 1970s [60], and with PAs being rated less favourably on all measures to monitor patients with diabetes and their patients less likely to achieve targets for disease control in 2002 [79].

#### Summary of evidence regarding the outcome of care

These studies highlight that the aspect of PAs’ impact that has been reported consistently is that of acceptability to patients and potential patients in family medicine settings in the USA, as well as to potential patients in a country where the PA role did not operate at that time [78]. There are some situations where they would prefer to see a doctor either for the complexity or severity of their condition, suggesting that patients who have experienced a PA or who envision such care feel they can determine which level of provider is appropriate for their need. The evidence on technically appropriate care provided by PAs, while mainly positive, is from often poorly reported studies, and there are also some less favourable comparisons with other providers. In addition, there is limited reported exploration of the appropriateness of care to patients who form the majority of the workload – the patients consulting with acute, undifferentiated conditions, or comparison with care provided by doctors for the same case mix of patients.

## Discussion

According to the literature presented, the number of PAs in family practice has increased over the profession’s 40-year history with approximately 50% working in family practice. Retention of PAs is considered possible if the conditions of the local area, as well as their employment, fit their personal circumstance. Clinician support for the profession is reported to be high, particularly amongst those already employing PAs, though some consider it to be a low salaried position. PAs are also considered to be expensive, because their work involves low revenue-generating patients. The apparent support for PAs, coupled with increasing numbers, appears to fit with a picture of need in terms of workload demand in family practice. The evidence for this comes in the studies that describe that the consultation type carried out by PAs is the acute, often undifferentiated caseload in family practice, with some suggestion however that the doctors see the older patients with more chronic or serious conditions. PAs are presented in several studies to potentially increase the workload of others through the need for supervision and (in the UK in particular) for prescribing support, though they may also enable an increase in physician or practice productivity. Acceptability to patients appears to be very high in actual and hypothetical situations, although it was reported that there were conditions patients would prefer to see a doctor for. Other reports on the outcomes of care are positive in the main, though limited, with surprisingly little on the appropriateness of the care provided for the major reported workload group of acute conditions. When summarised against the contemporarily used three dimensions of quality – patient safety, effectiveness of care and patient experience - in the UK NHS [80], the review suggests that some supportive evidence for the PA in general practice has been found in each of these dimensions, albeit in limited form outside of patient experience. However, there are a number of caveats to the support regarding patient safety and effectiveness of care as the findings do not provide robust evidence and there is a complete absence of studies in some areas.

The second key message from the review is the issue of context and method. The majority of the studies included are from the USA, reflecting the development of this professional group since the 1970s and its relatively recent introduction in a small number of other countries. Most of the studies are of weak to moderate quality as assessed against critical appraisal checklists. Quantitative descriptive studies with no, or limited, comparative data dominate the literature. Where comparative data are presented, contextual factors, potentially confounding any analyses, are only controlled for in modelling studies. Qualitative studies rarely described their methods and analysis thoroughly. Although methodological strength and reporting quality (particularly of study methods) were considered to have improved over the 40 year period of the literature included in the review, large numbers of the studies were from the 1970s. It might be assumed that the concentration of studies from the 1970s reflected interest (and potentially attempts to promote the role through local evaluation) in what was then a new occupational group in the USA. However, the apparent lack of change in research questions over time was more surprising, particularly in light of remaining gaps in the literature. This is exemplified in the limited reported exploration of the appropriateness of care to patients who form the majority of the workload – the patients consulting with acute, undifferentiated conditions - or comparison with care provided by doctors for the same case mix of patients. It might be that the slowing down of evidence production alongside the growth in PA numbers can be seen as acceptance of the contribution of PAs as an occupational group. However, these issues of context and method limit the generalisability of the findings to PAs in family practice not only within the USA but also to the newly developing roles in the UK, Netherlands and Australia. Notwithstanding the low quality there is reasonable consistency of findings, particularly regarding conditions seen and acceptability of the role.

The implications of the findings of this review are twofold: the implications for the development of the PA role in primary care in the UK and countries with similar clinical primary care systems, and those for further research. The way in which the PA role is being utilised (and developed) in the UK is very much in line with the way of working in the USA, focusing on acute presentations or ‘same day’ workload in general practice [17]. The literature suggests that this use of the role may enable doctors to focus on complexity in their caseload, utilising their training and experience while PAs deliver care that might be considered more straightforward, but at a level that is acceptable to both patients and the PAs’ employing doctors. If the findings from the USA are replicable it is also possible that PAs might fill any geographical gaps in the medical primary care workforce. This niche for PAs is identified in a small qualitative study of employers of PAs in the UK [16]. Despite 40 years of studies, the evidence pertaining to PAs in family practice remains descriptive and weak, accentuated by poor reporting. This issue has not been addressed in previous reviews, but is acknowledged in a recently published review [25]. This lack of evidence does not appear to be unique to the PA role. A dearth of evidence is reported about changing workforce skill mix, especially for role changes out with doctors and nurses, and most particularly a lack of evaluation of cost effectiveness and impact on the wider health care system [81]. As changing workforce skill mix is a strategy in use to improve effectiveness and efficiency of healthcare, good research evidence is needed about the likely consequences of any skill mix change [81]. While our review of PAs in general practice settings provides some evidence of the consequence of the change from doctors to PAs, the review also makes it clear that a number of research questions remain, in general and in relation to primary care in the UK and similar clinical primary care elsewhere. The questions we consider merit further investigation are:

• What is the volume of PAs as part of the total primary care workforce in the USA or elsewhere?

• What motivates PAs to work in primary care?

• What impacts on other human resource aspects such as retention and turnover rates? What is the efficiency of the shift in work between professional groups?

• What is the impact of contextual factors in the work place, for example practice configuration, PA experience, expectations of others on the activity, productivity and outcomes of PAs?

• What is the value in terms of health outcomes of re-directing doctors’ time to patients with complex, chronic conditions (and away from patients with relatively minor, self limiting conditions)? What is the economic cost and benefit of PAs in primary care?

• How appropriate is the care to patients who form the majority of the PAs’ workload – the patients consulting with acute, undifferentiated conditions?

We suggest that these are questions that warrant further, country specific, investigation in good quality studies providing comparative data with other relevant professional groups. In this way, health service planners, managers and commissioners might be provided with evidence to support their decision making as to the best deployment of their finite resources.

This review has a number of limitations. Firstly, the review had a specific question, focusing on primary care as relevant to the UK and European definition of primary care, that is, care provided in general practice. This tightly defined focus together with the exclusion of studies where the primary care data could not be disaggregated from data in the secondary care setting [82] may have limited the available evidence. However, this approach has assisted in identifying the evidence as relevant to those countries with similar primary care systems to the UK in which PAs are starting to be employed and even trained, even though the setting for the majority of the studies was the USA. The approach can also aid those trying to transfer knowledge about workforce issues from one health care system to others. Secondly, the review included many studies that might be considered outdated being from the 1970s and 1980s and therefore being carried out when family practice in the USA (and indeed elsewhere) was more dominated by single handed physician practices prior to the move to the health managed organisations or other group practice configurations more prevalent today. This historical contextual change potentially limits the generalisability of the findings. The age of the studies is also relevant in that standards of research reporting were less rigorous at that time and the several studies with limited descriptions of method, for example, limit the opportunities for synthesis of findings and the strength of conclusions that can be drawn. However, the rationale for the inclusion of such studies rested on the fact that the PA role is only recently introduced in many countries and findings from the early phases of role development in the USA may therefore be highly relevant. The fact that the vast majority of the literature is from the USA might also be seen as an interesting finding of itself, particularly as other countries are appearing willing to at least trial the PA role without health system-specific evidence. There appears to be a progression in the reporting of new workforce roles which moves from the descriptive to single site evaluations to multi-site evaluations [83,84] and to ignore this would diminish the evidence for those considering introducing new roles. Interestingly, no dramatic changes in findings are reported in the included studies over the 40 year period or in the papers from settings outside of the USA, potentially suggesting that issues of context and method are not a complete barrier to the usefulness of the evidence presented in this review. Lastly, the review only included published studies and did not include any grey literature. While the value of the inclusion of unpublished literature in meta-analysis of randomised control trials has been established [85] it has not been established for a narrative review such as this whether the absence of unpublished empirical studies biases or detracts from the overall conclusions. However, there may well be unpublished reports, particularly those internal to health care providing organisations, and of a commercially sensitive nature, which provide further evidence.

## Conclusions

The evidence of the contribution of PAs to general practice as a subset of primary care, mainly in the USA, was mixed in its findings across a range of measures constituting ‘contribution’ and somewhat limited in its generalisability. The evidence regarding structure indicated the USA experience has been one of growth in numbers of PAs working in primary care over thirty years, indicating a tacit, positive, view of the value of PAs as a new health provider role to some employers in meeting demand for health care. In terms of other processes and outcomes, their acceptability to both patients and professional colleagues is repeatedly reported. There is some indication that this positive experience of contribution to the workforce is being replicated outside of the USA. However, the published evidence is, in the main, of weak or moderate quality and with little that provides comparative or economic analysis to help inform decision makers looking for strong research evidence, particularly outside of the USA in countries where the PA role is much earlier in its development, as to the potential benefits of the PA role as either a substitute or complement to other mid level roles and/or physicians, in addition to the strong inferred endorsement of the role. A number of questions merit further investigation to assist those making choices in which staff to employ to best meet the needs of their patient population within finite resources and in their own health system.

## Competing interests

The authors declare that they have no competing interests.

## Authors’ contributions

MH, VMD, SdeL, JG, HG designed the review. MH, KC, WC, JY, VMD, SdL and HG screened titles and abstracts and evaluated article inclusion and study quality. MH wrote the first draft of the manuscript. All authors revised for important intellectual content and approved the final manuscript.

## Pre-publication history

The pre-publication history for this paper can be accessed here:

http://www.biomedcentral.com/1472-6963/13/223/prepub

## Supplementary Material

Additional file 1 Studies of structure – data extraction. Click here for file

Additional file 2 Studies of process – data extraction. Click here for file

Additional file 3 Studies of outcome – data extraction. Click here for file
