# Supplementary material for: The contribution of Physician Assistants in primary care: a systematic review
Source: BMC Health Serv Res. 2013 Jun 18;13:223. doi: 10.1186/1472-6963-13-223 (PMC3698179; doi:10.1186/1472-6963-13-223)
Supplement: Additional file 1 — Studies of structure – data extraction. [file 1472-6963-13-223-S1.docx]

Additional file 1

| **Reference number** | **First Author**  **Year**  **Study Design** | **Aim** | **Study population**  **Setting (including country)** | **Sample** | **Key findings** | **Major strengths and limitations** |  |  |
| --- | --- | --- | --- | --- | --- | --- | --- | --- |
| 10 | Scheffler, RM  1982  Survey | To estimate demand for PAs employed by physicians | Active, non-federal, office based, patient care physicians  Physician Master File of the American Medical Association. Pan USA (excluding Alaska, Hawaii and US possessions) | a) Postal- National probability sample out of 181,182 physicians-stratified and simple randomisation- 6092 physicians  Response rate- 51% (3076)  b) Telephone- stratified and randomised sample size- 200 physicians who did not respond to the postal survey | **Physician support**  Estimated number of PAs to be hired by physicians, 1976:  19.5% in GP/family practice (23.5% on conditional trial)  Physicians expected to pay PAS a lower salary than was being received in the market place:  Expected annual salary (in US$) to be paid to PAs just completing training by physicians, by willingness to hire PAs, 1976  a) Will hire- n=63 (mean salary-$12127, SD 3055)  b) Will not hire- n=167 (mean salary- $12293, SD 4741)  c) Will hire trial only- n=29 (mean salary-$11400, SD 3256)  d) Total- n=259 (mean salary- $12153, SD- 4235).  Those who already hire PAs are more likely to hire again. | Good quality, using large sample and regression model.  Willingness to hire concept may not reflect actual practice |  |  |
| 32 | Oliver, DR  1977  Survey | To measure the level of success of Iowa programme graduates employed in Iowa as PAs. | Type-A PAs employed in Iowa state, USA | Sample size not stated | **Numbers of PAs**  61% (36) of primary care PAs were working in general/family practice: | Methods and sample size not described |  |  |
| 33 | Larson, EH  1994  Survey | To discover and describe the differences in personal characteristics and practices of MEDEX graduates in rural areas compared with those in urban areas. | MEDEX Northwest graduates (PA training programme at the University of Washington), USA | 425 PA graduates- 341 traceable (with address). Response rate: 86.5% (295). 241 PAs who completed the survey and practices in the USA. | **Numbers of PAs**  67.5%, 54.3% and 58.5% of primary care PAs were working in rural, urban family practice and in total, respectively. (Difference significant at α =0.10 level) | Good quality study, with high response rate.  Study of one training programme only, with limits to generalisability |  |  |
| 34 | Hooker, RS  1991  Survey | To describe how PAs are used in a large group practice health maintenance organisation | PAs in primary and non-primary care at Kaiser Permanente Northwest Region, USA, 1990 | 65 PAs | **Numbers of PAs**  49% of PAs were working in family practice | Descriptive paper.  Unable to distinguish family practice in most of the paper  Study of one HMO only, with limits to generalisability |  |  |
| 35 | Perry, HB  1977  Survey | To describe the background characteristics, the work environments, and job characteristics of PAs | 1,282 PAs (virtually the entire PA profession as it existed at that time – not further specified). Graduated in 1973 or 1974  Throughout USA, late 1974 and early1975 | 939 completed surveys (73% response rate) | **Numbers of PAs**  Specialty of PA known for n=902: 29.1% (n=262) in family practice and 14.5% in general practice | Pan USA.  Descriptive paper.  Unable to distinguish family practice in most of the paper. |  |  |
| 36 | Perry, HB  1981  Survey | To develop and maintain current information for the PA profession | PA graduates (member programmes of the Association of PA Programmes), USA | Response rate-63% (4500 PAs); Eligible PAs (still in practice as a PA)- 3416 | **Numbers of PAs**  52% of respondents were working in family/general practice. Since 1974, there appeared to have been a shift within the profession towards employment in family practice. | Pan USA.  Unable to distinguish family practice in most of the paper. |  |  |
| 37 | Muus, KJ  1996  Survey | To obtain insights into similarities and differences between rural and urban PA practice across a range of issues. | Random sample of 2500 PAs drawn from the AAPA national membership database, USA | Response rate: 62.5% (1560 PAs) | **Numbers of PAs**  31.7% in Family medicine | Pan USA (although AAPA members only)  Unable to distinguish family practice in most of the paper  Difficult to follow figures in tables from text. |  |  |
| 38 | Pan, S  1997  Survey | To compare similarities and differences between primary care nurse practitioners (NPs) and PAs | PAs and NPs practicing in primary care related fields  PA data: national survey of American Academy of PAs (AAPA) members  NP data: 1992 National Survey of Certified NPs and Clinical Nurse Specialists  USA | PAs- 667  NPs- 1091 | **Numbers of PAs**  In family medicine:  PAs- 486 (31.7%)  NPs- 212 (15.4%) | Pan USA (although AAPA members only)  Unable to distinguish family practice in most of the paper  PA and NPs compared from two different survey datasets | |  |
| 39 | Duryea, WR  2000  Survey - secondary data analysis | To analyse the practice profiles of PAs 60 years of age and older- many of whom are approaching retirement. | Includes both AAPA member PAs and non-member PAs who are eligible to practice as PAs in the USA  1998 AAPA Census- 21,755 members and 14,143 non-members were polled | Not stated | **Numbers of PAs**  In family practice/general medicine: a) All PAs- 5,135 (39.6%) b) PAs aged 60 years or more by the end of 1998- 92 (46.5%). Highest among clinical specialties | Pan USA (although AAPA members only)  Secondary analysis – cannot check question definitions  Unable to distinguish family practice in most of the paper  Relatively small numbers of PAs over age 60 makes comparisons limited |  |  |
| 40 | Larson, EH  2001  Survey -Secondary date analysis | To estimate the relative productivity of PAs compared to physicians | National -AAPA data, USA Individual-level data from American Academy of PAs (AAPA), 49,641 graduate PAs in 2000 in the AAPA data set | Not stated | **Numbers of PAs**  72.8% (2891) and 56.3% (2236) of PAs were working in family/general medicine in 1991 and 2000, respectively (Only PAs active in both 1991 and 2000 for whom speciality was known were included). | Pan USA  Much lower response rate for non-AAPA members | |  |
| 41 | Hooker, RS  2002  Survey - secondary data analysis | To summarise trends in the supply and education of PAs and NPs. | All PAs 52,716 graduated from an accredited PA programme by 2001 (45,120 employed as PAs) and NPs studying or practicing in USA (102,829 NPs with education, 58,512 in NP role). | Sample size not stated. | **Numbers of PAs**  34.5% family practice / family, {NPs 36.0%} | Pan USA  Method only describes source of data.  PA and NPs compared from two different survey datasets | | |
| 42 | Oliveria, SA  2002  Survey | To determine physician use and amenability to use of non-physician health care providers to perform skin cancer screening in comparison with other cancer screening examinations. | All (647) family physicians practicing in this setting- List obtained from the Illinois Academy of Family Physicians (IAFP) in 2000  Predominantly rural, southern three-fourths of Illinois, USA | Response rate: 35% (n=226). | **Physician support**  Incentives to PA use: reduce patients wait for appointments (90%), relieve physician’s workload (86%), increase the practice’s productivity (84%), permit more patient education/counselling (78%), allow physician to spend more time on complex cases (72%), increase patient satisfaction (71%)  Unreceptive physicians identified 4 constraints: opposition from patients (62%), increased malpractice risk (59%), PAs tend to overstep their authority (52%), loss of continuity of care with physician’s patients (52%). | Large pan-USA study.  Response rate relatively low.  Unable to distinguish family practice in most of the paper |  |  |
| 43 | Larson, EH  2007  Survey - secondary data analysis | To describe key elements of change in the demography and distribution of the PA population between 1967 and 2000, as well as the spread of PA training programmes. | 49641 graduate PAs in 2000 in the AAPA data set | 49,641 | 72.8% (2891) and 56.3% (2236) of PAs in family/general medicine in 1991 and 2000, respectively (see also Larson 2001) | Pan USA  The data distinguishable by family practice in this paper are a repeat of Larson, 2001. |  |  |
| 44 | Duttera, MJ  1978  Observation | To describe the practice activities of physician assistants and their physician mentors and to describe their working inter relationships | Physician Assistant graduates from a two tear programme working continuously in rural primary care practice for more than six months in 14 practice sites  Six south eastern states, USA | 788 outpatient-provider encounters | **Retention**  PA-physician relationship was well established, 14 out of the 17 PAs were working in the same practice for 1 year or more. | Selection of these 17 sites (from national survey) not specified.  Observational study may not be large enough to capture all variation. |  |  |
| 45 | Henry, LR  2007  Qualitative interviews and focus group | To determine the factors that influence autonomous rural PAs who work < 8 hrs per week with their supervising physician) to remain in remote locations | Community residents of Texas rural towns, USA.  PAs identified through snowball sampling via PA organisations, educational establishments.  PA working autonomously < 8 hours contact with supervising MD. PA sole primary care practitioner in community. PA worked > 24 months in community. Town < 4,000 residents. No other primary health care options within 25 mile radius | 8 towns, 8 PAs, 8 interviews and focus groups | **Retention**  1) confidence in the ability to provide adequate health care 2) desire for small town life 3) residing in the community 4)being involved with the community. | Well described study.  Limited to one USA state, with potential self-selection bias of participating practices |  |  |
| 46 | Joiner, CL  1974  Survey | To determine the attitudes of primary care physicians toward PAs. | Random sample of 450 Alabama state primary care physicians. Sample represented 53 of the 67 counties and 92 cities/townships within Alabama, USA | Response rate - 68.9% (310); 45.5% (141) of primary care physicians were working in general practice. | **Physician support**  70.2% of general practice physicians were in favour of PA concept; 26.3% were not in favour of PA concept; 3.5% were not sure (undecided). | Brief report with limited description of method and respondents. |  |  |
| 47 | Oliver, D  1980  Survey | To examine the attitudes of physicians who employ PAs, family practice residents and sophomore medical students to PAs, and the perceptions of PAs about their role | Iowa physicians employing a PA, Iowa practicing PAs employed by family physicians, sophomore medical students, family practice residents.  Iowa, USA; small communities (average size+5000-10000); family practice; employed a PA for minimum of six months | 30 physicians employing a PA, 43 PAs, 167 family practice residents (54 at lst yr , 41 at 2^nd^ year, 34 at 3^rd^ year, 18 recent graduates and 20 staff physicians). | **Physician support**  Responses for the four groups in this order – physician employer, PA, residents, students.  Need for PA in rural areas – 100%, 100%, 87%, 93%; in urban areas 86, 93, 73, 81; suburban areas 79, 78, 53, 65%.  Quality of PA training excellent 76%, 41, 10, 21; good 24, 52, 51, 37. Services rendered by PAs extremely useful 75%, 65, 14, 35; useful 25%, 35, 74, 59%  Situations that would warrant hiring a PA: patient load large enough 70, 70, 54, 77; no other physician available 47, 40, 64, 75; improve quality of care 57, 65, 47, 62%, alleviate on-call 50, 35, 29, 54%.  A PA would increase number of patients cared for (93, 95, 97, 97), allow more physician leisure time (63, 77, 64, 75); allow more comprehensive care to patients (97, 98, 83, 88); allow more time for complex tasks (100, 91, 94, 95), enable more home/nursing home visits (75, 65, 79, 82%). | Included three different groups of ‘physicians’.  Well described except study population size and response rate.  Limited to one USA state |  |  |
| 47 (continued) | Oliver 1980 (continued) |  |  |  | **Structural barriers**  Jeopardise physician-patient relationship 17, 5, 32, 23%; decreased profits 17, 7, 4, 6%; lower quality of care 3, 2, 12, 6%; higher probability of malpractice case 23, 9, 43, 22%; depersonalisation of physician’s role in patient care 40, 23, 58, 47%. |  |  |  |
| 48 | Ford, VH  1998  Qualitative interview | To examine the perception of family physicians towards nurse practitioners and physician assistants. | Family practice 3 year residency program in Southeast USA linked with a medical school – faculty and resident staff  South East USA | 10 interview: five faculty and five residents | **Physician support**  Overall positive feeling towards both NP and PA, but limited experience of working with either role. Could describe PA educational requirement (described as a medical model) but half could not describe for NP (described as a nursing model). Happier for PA prescribing compared to NP prescribing, some fear of PA / NP setting up independent practice.  Both NP and PA described as taking more time and being more thorough in obtaining patient histories than physicians.  Opinion of respondents was that NPs and PAs were cost-effective. | Interview (topic) guide is not described.  No respondent transcript excerpts are used to support thematic analysis.  Limited to one university where authors acknowledge NPs had not been trained but PAs were active contributors. |  |  |
| 49 | Engel, GV  1984  Survey | To determine the future hiring intentions of physicians with regard to PAs and their reasons | All physicians (*n*=72) on three Family medicine residency programmes in southern California, USA.  58% had been familiar with the PA occupation and had worked or trained with PAs prior to FP residency | 55 returned surveys | **Physician support**  61% (*n*=44) respondents would hire a PA in their future practice. Four answered negatively, seven undecided.  Two main reasons for hiring a PA were reported – ‘to gain more control over work hours’ (*n*=21) and ‘opportunity to expand patient care’ (*n*=23). Would most like to see PAs carrying out patient education.  **Structural barriers**  Factors that might deter the hiring of a PA were administrative responsibilities (10 of those who said they would consider hiring) and malpractice considerations (all those who had said no or undecided re- hiring) | Limited description of method.  Limited to one geographical region. |  |  |
| 50 | Isberner, FR  2003  Survey | To assess physicians' attitudes, experiences, and practices regarding the utilisation of PAs | All (*n*=647) family physicians practicing in predominantly rural, southern three-fourths of Illinois, USA. List obtained from the Illinois Academy of Family Physicians (IAFP) in 2000 | Response rate: 35% (*n*=226); with n=56 in the lowest quartile (negative regarding PAs) and a random sample of n=56 of the highest quartile (very positive towards PAs) used for analysis | **Physician support**  Receptive physicians reported 6 incentives:  Reduce patients wait for appointments (90%), Relieve physician’s workload (86%), Increase the practice’s productivity (84%), Permit more patient education/counselling (78%), allow physician to spend more time on complex cases (72%), increase patient satisfaction (71%)  **Structural barriers**  Unreceptive physicians identified 4 constraints: opposition from patients (62%), increased malpractice risk (59%), PAs tend to overstep their authority (52%), loss of continuity of care with physician’s patients (52%).  Receptive physicians also identified perceived patient opposition as a constraint (48%). | Well described study.  Analysis includes only responses from those most or least in favour of PAs  Limited to one geographical region. |  |  |
| 51 | Sells, CJ  1975  Interviews | To examine in detail the activities of a small number of representative MEDEX graduates regarding their activities in provision of child health services. | 58 Medex graduates from first 4 MEDEX classes working with FPs in rural communities in Washington State, USA. PAs employed for at least 9 months prior to study | Six interviews | **Physician support**  Physicians liked the MEDEX and felt they were well accepted by the patients. | Method for interview and analysis poorly described. No interview excerpts used – presents generalised statements only.  Limited to one geographical area. |  |  |
| 52 | Drass, KA  1988  Observation with field notes | To assess whether nurse practitioners use interactive strategies which are different from those employed by physician assistants during encounters with patients. | Mid-level providers in an adult medicine ambulatory clinic in a federally qualified health maintenance organisation in a large Midwestern city, USA | Two PAs (1 male, 1 female), one family nurse practitioner (female). | **Nurse support**  Nurse practitioner perceives that NPs are better prepared for mid-level provider role than PAs because most patients are ‘worried well’ and NPs provide better support to these people than PAs who are more concerned with medical problems. In contrast PAs thought that NPs less qualified for mid-level role. | Field note data only used to elicit these data.  Very small sample in one HMO (unclear how/why this selection was made) |  |  |
| 53 | Parle, J  2006  Interviews, focus groups | Evaluate the impact of an initiative to recruit US-trained PAs | Up to nine general practices employing PAs in West Midlands, UK | Not specified | **Physician support**  PAs well received by doctors at all levels, practice nurses, non-clinical staff (and patients).  Appreciated PAs’ enthusiasm, interpersonal skills, approach to patients, communication skills, flexibility, patient documentation, team working. | First published UK data.  Method and sample for interviews and focus groups is not described. No interview excerpts used – presents generalised statements only. |  |  |
| 54 | Dehn, RW  1995  Survey | To rank the impediments to practice as perceived by PAs in Iowa. | All 292 PAs practicing in Iowa, USA | Response rate- 78% (220). Usable study population- 210 PAs (as 10 PAs were not practicing). 68.1% (143) of Iowa PAs were working in family practice | **Structural barriers**  Perceived impediments to PA family practice in Iowa (ranking):  1) Third-party payment limitations - Family practice- 44.8% (64); not family practice- 20.9% (14); p<0.0009  2) Medicare payment limitations. Family practice-28% (40); not family practice- 17.9% (12); p<0.12.  3) Hospital and clinic regulations. Family practice- 7% (10); not family practice- 38.8% (26); p<0.00001 (statistically significant). 4) Medical community resistance. Family practice- 7% (10); not family practice- 4.5% (3); p<0.48.  5) Federal regulation restrictions Family practice- 4.2% (6); not family practice- 10.4% (7); p<0.08.  6) Iowa state regulations. | Adequately described study.  Limited to one USA state |  |  |

Studies of STRUCTURE: Numbers of PAs working in primary care, retention of PAs in general practice, physician support and willingness to employ PAs, structural barriers to the employment of PAs
