# Supplementary material for: The contribution of Physician Assistants in primary care: a systematic review
Source: BMC Health Serv Res. 2013 Jun 18;13:223. doi: 10.1186/1472-6963-13-223 (PMC3698179; doi:10.1186/1472-6963-13-223)
Supplement: Additional file 2 — Studies of process – data extraction. [file 1472-6963-13-223-S2.docx]

**Additional file 2**

| **Reference number** | **First Author**  **Year**  **Study Design** | **Aim** | **Study population**  **Setting (including country)** | **Sample** | **Key findings** | **Quality assessment (major limitations)** |
| --- | --- | --- | --- | --- | --- | --- |
| 38 | Pan, S  1997  Survey | To compare similarities and differences between primary care nurse practitioners (NPs) and PAs | PAs and NPs practicing in primary care related fields, USA  PA data: national survey of American Academy of PAs (AAPA) members.  NP data: 1992 National Survey of Certified NPs and Clinical Nurse Specialists. | PAs- 667  NPs- 1091 | **Activity levels/throughput** Average number of outpatient visits/week in family medicine: PAs- 105; NPs- 75 | Pan USA (although AAPA members only)  Unable to distinguish family practice in most of the paper  PA and NPs compared from two different survey datasets |
| 41 | Hooker, RS  2002  Secondary data analysis | To summarise trends in the supply and education of PAs and NPs. | All PAs 52,716 graduated from an accredited PA programme by 2001 (45,120 employed as PAs) and NPs studying or practicing in USA (102,829 NPs with education, 58,512 in NP role). | Sample size not stated. | **Activity levels/throughput** Average number of outpatient visits per week in family practice: PA 105; NP - 75 | Appears to present same data as Pan, 1997 |
| 44 | Duttera, MJ  1978  Observation | To describe the practice activities of physician assistants and their physician mentors and to describe their working inter relationships | Physician Assistant graduates from a two tear programme working continuously in rural primary care practice for more than six months in 14 practice sites  Six south eastern states, USA | 788 outpatient-provider encounters | **Patient consultation types** House calls and nursing home visits in five practices.  Minor trauma (48 cases observed) and administrative physical examinations for employment, insurance or school (32 cases observed).  In case of triage, PAs managed patients with less serious problems than those seen by the physician (χ²=22.75, p<0.001). When patients were not assigned in a planned manner, PAs managed the more severe problems (χ²=16.99, p=0.03). | Selection of these 17 sites (from national survey) not specified.  Observational study may not be large enough to capture all variation. |
| 48 | Ford, VH  1998  Qualitative interview | To examine the perception of family physicians towards nurse practitioners and physician assistants. | Family practice 3 year residency program in Southeast USA linked with a medical school – faculty and resident staff  South East USA | 10 interview: five faculty and five residents | **Patient consultation types** Both NP and PA attend “common” illnesses, chronic illnesses and acute problems such as otitis media. | Interview (topic) guide is not described.  No respondent transcript excerpts are used to support thematic analysis.  Limited to one university where authors acknowledge NPs had not been trained but PAs were active contributors. |
| 51 | Sells, CJ  1975  Observation (time and motion) | To examine in detail the activities of a small number of representative MEDEX graduates regarding their activities in provision of child health services. | 58 graduates from first 4 MEDEX classesworking with FPs in rural communities in Washington State, USA. PAs employed for at least 9 months prior to study | 6 graduates | **Patient consultation types** 30% of total time with patients (37% paediatrics); paperwork 15% time. Diagnostic categories by time – well-child examination 30%, minor surgery 21%, respiratory illness 11%, orthopaedics 9%, dermatology 6%, ENT 5%, ill-defined 5%, GU 4%, allergy 4%, GI 2%.  **Activity levels/throughput** Average 8 paediatric and 11 adult office patients seen per day. During winter months 20% more are seen. Average time 10 minutes per child, 11 per adult. | Method for observation described briefly; unclear how the 6 PAs were selected.  Limited to one geographical area. |
| 53 | Parle, J  2006  Medical record review | Evaluate the impact of an initiative to recruit US-trained PAs | Three general practices inc. 7 PAs, 4 GPs, 2 NPs and 2 practice nurses  West Midlands, UK  Nine general practices | Not specified | **Patient consultation types** Classification of condition:  General and unclassified PA 16%, GP 38%, NPs 36%, nurses 59%.  Digestive PA 5%, GP 8%, NPs 0%, nurses 0%  Circulatory PA 9%, GP 7%, NPs 11%, nurses 6% / Musculoskeletal PA 13%, GP 14%, NPs 0%, nurses 0%  Psychological PA 11%, GP 12%, NPs 0%, nurses 0%  Respiratory PA 17%, GP 13%, NPs 28%, nurses 9%  Skin PA 10%, GP 8%, NPs 9%, nurses 0%  Endocrine, metabolic, nutritional PA 7%, GP 0%, NPs 10%, nurses 17%  Pregnancy, childbearing, family planning PA 5%, GP 0%, NPs 0%, nurses 0%  Female genital PA 7%, GP 0%, NPs 6%, nurses 9%.  **Activity levels/throughput** PAs average 16.5 consultations per day; GPs 17 per day.  8/9 practices had increase in patient list size by 2.4-5.3% in one year  **Impact on work of others**  PAs taking some responsibility for tasks previously performed by GPs e.g. checking all investigation results. | First published UK data.  Method and sample are not described. |
| 53 (continued) | Parle, J (continued) |  |  |  | **Support and supervision**  36-68% of all contacts from PA to GP about a particular patient to seek signature on prepared prescription; 1-16% to review treatment plans. |  |
| 55 | Golladay, FL  1973  Survey and modelling | To analyse the potential impact of physician extenders on the productivity of primary care practices | Urban and rural, group and solo practices in Wisconsin, Vermont, North Carolina, USA  Two practices employing PAs. Other personnel include; MD, registered nurse, licensed practical nurse, medical assistant, x-ray technician, laboratory technician. | Not specified | **Patient consultation types** As practice expands PA assumes responsibility for; treating warts (and follow-up), removing ear wax, treating sore throats, treating sinusitis, performing well-child examinations, treating minor burns, treating muscle contusions (without x-ray).  **Impact on workload of others**  Potential productivity of individual physician could be increased by 74% by using a PA. Efficient practice could care for 147 patients using conventional med workers, introduction of PA would enable it to have 265 patients. | Detailed description of the modelling activity but scant detail on initial data collection for use in the model.  Authors note limitations to the model, e.g. not does allow for random arrival of patients’ complaints and used data from one practice only. |
| 56 | Miles, DL  1976  Medical record review (before and after study) | To examine certain consequences of using PAs on the functioning of a primary care system in a medically underserved, rural community. | Six physician practices in rural, economically depressed county of approx 27,000 inhabitants in Southern Appalachia, USA. County served by 6 physicians in 1971 (22 per 100,000). Late 1971 5 PAs employed in 5 of the 6 offices, by time of study (three years later) 3 remain. | Not specified | **Patient consultation types** Of the patients seen by a PA 73.5% were NOT seen by the physician. Proportion of patients seen for acute, chronic and preventive reasons is nearly identical when comparing combinations of physician and nurse with PA and nurse (acute; 60.5% vs 62.5%, chronic 21.6% vs 24.9%, preventive 17.9% vs 12.6%).  **Activity levels/throughput** PAs  Utilisation increased by one third six months after PAs introduced. 121 patient visits per week baseline (July 1971), 166 visits per week Feb 1972.  **Use of other healthcare services**  Physician / PA teams hospitalised increasing proportion of patients, use of PAs increased tendency to hospitalise insured versus uninsured patients. | Well described study, with measurements at three points over time.  Authors accept that some assumptions about PAs substituting for physicians made on basis of overall increase in overall utilisation, while other explanations are possible.  Limited to three practices in one geographical area. |
| 57 | Rushing, WA  1977  Medical record review and survey | To examine the effect of the New Health Practitioner, specifically PAs on socioeconomic inequities in one community. | Patients attending the 3 clinics that had MD/PA team operating for whole of study period.  Economically depressed county in Southern Appalachia, USA.  1970 pop approx 27,000, 6 full time physicians. 5 PAs employed in 6 practices, all male, graduates of MEDEX, employed in same practice where did clinical training | Estimate 2300 based on results presented being 74.6% of total sample. | **Patient consultation types** Physicians see more white patients of higher socioeconomic status compared to the PA. The relationship between physician and higher status patients (race combined) remains for all types of care; acute, chronic and preventive; for age; over time and when type of payment is controlled. | Reports from the same study as Miles, 1976 (54).  This subset analysis is adequately described, but authors acknowledge small numbers in subset analysis.  Limited to three practices in one geographical area |
| 58 | Nelson, EC  1977  Work logs, medical record review and structured observations (before and after study) | To compare and contrast the role of the MEDEX with that of the physician | 11 primary care practices in rural New England, USA.  Run by 4 GPs and 7 internists with general practices in 3 solo, 4 partnership and 4 moderate sized group practices. PAs graduated from New England Medex programme in 1974 | Logs – 9 physicians. Patient encounter forms – 1132 patients  Observations – not specified. | **Patient consultation types**  PA acute illness 55% (Physician 37%), chronic 20% (physician 41%).  PAs more younger patients (under 44) and physicians more older patients 65 and over.  Frequency of five leading diagnoses – PA upper respiratory tract infection 11.7% (physician 9%), essential hypertension 7.5% (physician 13.8%), no abnormality 7.5%, obesity 4.9% (physician 4.5%), anxiety 2.8%; physicians arteriosclerotic heart disease 6.5%, diabetes 5%.  Direct contact time 49% (physician 48%), 33% documentation and analysis related to patient visit, 7% consulting with physician.  **Impact on workload of others**  Physician seeing more chronic illnesses (especially diabetes) and fewer patients with no abnormalities, and more older patients. Spends less time in direct patient tasks (68% reduced to 48%), reduced number of patients seen every hour (2.5 to 1.9), reduced average time spent with patient (16.3 to 13.9 minutes), increased time counselling patients (22 to 27%); increased indirect tasks (32 to 52%, including increase from 5 to 11% for supervisory matters). | Adequately described methods and sample, excepting missing information on the number of observations.  Descriptive statistics only presented for before and after periods, with no comparative analysis, with some concern about the conclusion regarding re-distributed physician activities. |
| 59 | Frame, PS  1978  Method not stated (medical record review likely) | To test whether outcome of diseases treated by the PA is equal to that of the supervising physician | Population - selected new diagnoses for 1975  Tri-County Family Medicine (non profit) providing primary care to rural area of western New York State, USA with medical staff of four FPs and one PA. A central practice and 4 satellites to which PA and physicians commute. PA graduate of 2 year New York programme. Receptionist decides if patient condition is in PA job description and offers choice to see PA (sooner than GP). PA in practice for 4 years. | Not specified  Formula devised by Nelson 1975: Total PA cost = direct PA costs + (Pa revenues/practice revenues x total overhead) | **Patient consultation types**  50% patients seen by PA, 50% by MD. Percentage of conditions seen by PA (remainder by MD): fractures 6%, chest pain 22%, peptic ulcer 25%, myocardial infarction 0%, URTI 76%, pharyngitis 75%, pneumonia 74%, oral contraceptives 77%, diabetes 44%, depression 47%.  **Support and supervision**  Immediate consultation with a physician for 8.4% cases; physician revisit arranged for 6.8%.  **Cost**  PA direct costs $12,400  PA revenue from direct patient visits $31,600  Satellite overheads $50,000: PA total costs $28,980 and small profit of $2,710 | Limited description of method and samples.  Limited to one practice setting, with restrictions on generalisability |
| 60 | Hill, RF  1979  Medical record review; observation, interviews | To probe parameters of feasibility of the PA role - utilisation, acceptance, quality of care and economics | Patients attending these clinics, PAs and other clinic staff.  Single PA-manned remote clinic (population 1239) in north central Oklahama, USA and three other private rural Oklahama clinics where PAs performed primary care services but the physician was not remote | Approximately  1,000 patient encounters randomly sampled at each site  over a 5-month period | **Patient consultation types**  Remote PA saw patients with a wide range of  ambulatory problems  Remote PA was more likely to see more general and less challenging problems as compared to control clinics  Remote PA's actions changed from 1^st^ to 2^nd^ year: Prescribing oral medication, advising and counselling patients, and performing urinalyses increased, but medical procedures, follow-up clinic appointments, referrals, cultures, and other laboratory procedures declined.  **Activity level/throughput**  Remote PA saw about 2 patients/hour - under-utilised and usage declined slightly during the 2^nd^ year as compared to the 1^st^ year  Control clinic PAs carried a patient load comparable to physicians in their respective practices  **Support and supervision**  Control clinic PAs handled about 98% of all primary care problems alone, (correlating well with the remote PA practice)  Approx. 25% rate of informal consultations between PAs and physicians in control clinics (mostly initiated by PAs) | Small study of one PA in one practice.  Methods and sample poorly described  Simple calculation of cost effectiveness, without analysis of repeat visits, physician supervision, etc |
| 60 continued | Hill, RF  1979  Medical record review; observation, interviews |  |  |  | **Cost**  Low revenue generation of Yale clinic: Operational costs exceeded revenues by about $7,000 during the 1^st^ year. The deficit increased during the 2nd year.  Remote PA was undercharging for services: Average fee at the 3 control clinics was almost $15 and at the Yale clinic was about $5 |  |
| 61 | Fethke, C.  1979  Observation | How are tasks allocated to the PA as a division of labour develops among personnel? | 19 physician supervisors and 28 PAs  Iowa, USA; 19 physicians employing 28 PAs in towns with populations of 50000 or less (14 PAs), towns of 5000 to 25000 (8 PAs) and 20000 or more (6 PAs). 11 group practices, 8 solo physician practices. Six PAs at satellite clinics away from supervising physician. 14 in family practice, five in primary care specialties | 1497 patients observed | **Patient consultation types**  Most frequent primary diagnosis – physical examination n=189, URTI 75, hypertension 58, otitis media 46, obesity 45, tonsillitis 38, perinatal care 34, ateriosclerotic heart disease 30, acute pharyngitis 25, UTI 24, dermatitis 23, warts 23, diabetes 23, bronchitis 22, well baby exam 21 i.e. a mix of acute and chronic presentations.  **Impact on workload of others**  PAs perform 68% of the tasks on the patients they see; 29% carried out by RN, 2.5% by MD. BUT RNs saw 80% of PAs’ patients for escort, explanation of examination, temperature taking or collection of specimens.  PAs saw patients of all levels of complexity and saw 23% of cases alone that met criteria requiring a consultation with a physician  **Support and supervision**  Very little interaction between physician and PA  – 126 patients (8.4%) | Detailed observation of PA activities.  Limited by descriptive statistics only, and being in one state of USA. |
| 62 | Larsen, KM  1982  Medical record review | To investigate the frequency and type of diagnostic and therapeutic procedures | All consultations for Oct 1979 and Jan, Apr, Jul 1980.  Small group practice in Anacortes, Washington, USA (population 10000), with two internists, three FPs and one MEDEX (9 yrs experience), with little selection of patient type by provider type. Catchment population 20000, 70 miles from tertiary care and nearest alternative medical care 20 miles away | 5698 patient visits (760 by MEDEX) | **Patient consultation types**  Diagnostic/therapeutic procedures on 592 patients (10%) – MEDEX 13.9%, internists 11.3%, FPs 7.5%. Top 10 procedures (99% of MEDEX procedures): ECP 0 (FPs 66%, Intern 80%), splint 29%, pulmonary function test 0%, suture removal 26%, sutures 12%, treadmill 0%, wart removal 35%, lesion removal/biopsy 1%, cast application and removal 17%, application of sling/collar 7%.  **Cost**  Differences in income generation by provider types – FPs performed 35% procedures and generated 34% income, MEDEX 22% procedures, 13% income, internists 43% procedures, 52% income. i.e. MEDEX = simple, time-consuming, disruptive less remunerative procedures | Limited to one PA and small group of physicians in a group practice.  Simple calculation of cost effectiveness, without analysis of repeat visits, physician supervision, etc |
| 63 | Martin, E  1984  Medical record review | To report the morbidity pattern encountered and temporal distribution of the problems after practice hours | All patient consultations for 32 nights on duty in Feb and March 1982  Rural Breckenbridge, Colorado, USA. Winter months extra evening clinic (5-10pm) to serve additional population in the ski season. Practice employing one FP, one PA and one retired surgeon; PA working alone in the after-hours service | 24 phone consultations and 174 patient visits to the evening clinic | **Patient consultation types**  Mean age 23, 15% <=5 years, 58% 16-35 years. Morbidity – four main groupings – infectious diseases 30.8% (61), musculoskeletal disorders 14.1% (21), trauma 11.6% (23), altitude-related illnesses 8.6%, otitis media 8.6%, vomiting and diarrhoea 8%, gynae 3.5%, opthamologic 3.5%, dermatologic 3.0%, cardiac 2.5%, miscellaneous 5.6%. Of these 40% (n=80) were considered to be emergent.  **Activity level/throughput**  Over the 32 days, an average of 5.6 patients (range 2.6 to 20) was seen during the day for each patient seen in the evening clinic  **Support and supervision**  Physician supervision involved chart review  within 24 hours of PA’s care (73% n=145);  telephone consultation at the time care rendered  12%, n=24), on-site participation when still on  site after 5pm (14%, n= 27) and physician call-in for 1% (n=2). | Adequately described study  Limited to one practice with specific patient seasonal caseload.  Simple calculation of cost effectiveness, without analysis of repeat visits, physician supervision, etc |
| 63 continued | Martin, E  1984 |  |  |  | **Cost**  Variable costs averaged $113 per night. Patients charged $23 for visit with a total average fee of $41, with total revenue of $7095. An average of 2.8 patients per night required tobreak even; average of 5.4 yielded a contribution of $3469 to fixed overheads. |  |
| 64 | Willis, JB  1986  Survey | To review data collected on practice speciality and performance of individual tasks by PAs | PAs in the 1984 American Academy of PAs (AAPA) Master file  USA | Random sample- 2719 PAs; Response rate- 52.7% (1433); 41.6% (596) of PA respondents were working in family/general practice. | **Patient consultation types**  Problems and diseases seen: a) PAs implement treatment- pulmonary, dermatologic, urgent care b) PAs formulate treatment plans- gastrointestinal, genitourinary, musculoskeletal, psychosocial, eye and ENT, cardiovascular, gynaecologic, hormone-related, renal, neurologic c) PAs establish working diagnosis- life support, neoplasia d) PAs gather data- neonatal.  PAs interpret data/results of bacteriologic studies and screening tests.  PAs defer to physician in ordering/performing- invasive tests, paediatric tests, rectal-lower GI procedures, allergy tests.  Perform minor surgery; rarely perform wound care, infection control or management activities. | Pan USA (although AAPA members only)  No explanation of the random sample size. |
| 65 | Mainous, AG  1992  Survey | To determine the extent of physician extender use and to explore patient factors associated with such use of physician extenders in primary care. | Adults (age 18 or older) living in Kentucky, USA  Random digit telephone dialling and interviewing | Response rate- 68.4%. Usable data-687 individuals | **Patient consultation types**  PAs were consulted regarding 36 different problems. “Cold” or “flu” and “check-up” each constituted 12% of the reported reasons for treatment. No other reason for visitation accounted for more than 10% of the reported problems. | Good population coverage with random selection.  Self report – unknown if respondents knew if seen a PA or NP  Limited to one state in USA |
| 66 | Dehn, RW  1999  Survey | To quantify how often various treatments and procedures were performed by PAs in family practice | Iowa, USA.74 PAs in single-specialty family practice | 55 surveys (74%) | **Patient consultation types**  (Scales for clinical activities of 0 never, 1 a few times a year, 2 at least once a month, 3 at least once a week, 4 daily.)  Provide patient education 3.95, dispense medication 3.44, make referrals direct to specialists 3.39, interpret radiographs 3.19, cryotherapy 2.92, contraception 2.91, manage depression by drug therapy 2.82, smoking cessation 2.80, repair/close lacerations 2.75, stress management 2.61, interpret electrocardiographs 2.60, manage depression by counselling 2.53, remove small skin lesions 2.43, psychological counselling 2.42, eye exam/foreign body removal 2.36, vision screening 2.33, use of microscope 2.15, incise/drain abcess 2.09; remainder less than 2.  Activities varied by size of population, with those in smaller communities carrying out more of the activities.  **Activity level/throughput**  Average number of patients per day 25 (15-60), daily inpatients 0.8 (0-10) and nursing home patients 4.2 (0-55). | Well described method.  Descriptive study in one USA state.  Author acknowledges limited ability to make conclusions by the small subsamples. |
| 67 | Grzybicki DM  2002  Medical record review | To measure the economic benefit of a family / general medicine PA practice | Family / general medicine practice offices in SW Pennsylvania, USA. PA serving population of 9,200 | 91 randomly selected patient records | PA saw younger patients with more acute conditions. Mean age PA patients 46 yrs, physicians 64  **Cost**  Mean charge for office visit - Physician - $45; PA - $47; NP - $47; Additional physician - $61  Same-task sub ratio of 0.86 compared with supervising physician. PA economically beneficial with compensation –to-production ratio of 0.36. Cost saving between $40,000 - $80,000 annually for a full-time PA | Well described study overall but no explanation of inclusion of the 91 cases only.  Limited study of one practice only.  Limited by assumptions of PA as direct physician substitute (does not consider appropriateness of care), and study of one practice only. |
| 68 | Simkens, ABM  2009  Medical record review (uncontrolled before and after study) | What differences were seen in contacts, morbidity, drug prescriptions and new referrals between the GPs and the PA? | All weekday patient contacts recorded by the PA and GPs One general practice in an urban, partly disadvantaged area in mid western Netherlands; 1.7 fte GPs, 1.8 fte practice assistants, 0.2 fte primary care nurse and 0.6 fte PA. USA- trained PA with two years' experience  Patient list size – 5096 in 2002, 5157 in 2005 | 7,837 GPs contacts 2002, 8025 GP contacts 2005, 1397 PA contacts 2005 | **Patient consultation types** PAs see more children (0-14); 22.4% of their workload) and 25-44 yr olds (40.1%) than GPs (12.7% and 28.9%).  Top ten diagnostic codes for PAs: acute URTI (3.1%), lower back symptoms (2.5%), cough (2.4%), dermatitis (1.8%), constipation (1.7%), allergic rhinitis (1.8%), dermaophytosis (1.6%), abdominal pain (1.5%), anxiety (1.5%), genital candiasis female (1.5%). GPs diagnosed more general and unspecified and circulatory problems; PAs saw more womens’ health problems.  **Activity level/throughput**  Number of patient contacts per wte PA = 2208; 60% of wte GP (3684 patients) | First study from the Netherlands in this review, and strengthened by use of before and after data.  Limited by studying only one PA in one practice, not being able to directly attribute any changes to the introduction of the PA (other factors not controlled) and only focusing on physicians rather than impact on any other clinician group. |
| 68 (continued) | Simkens (continued |  |  |  | **Impact on workload of others**  Number of GP contacts per wte GP decreased 3770 to 3684 2002-05 (2.3%); per wte PA 2208 (41% fewer than GP).  GPs’ workload changed significantly –more men, more patients aged over 45, more telephone contact and drug prescription/repeat. Type of diagnosis by GP also changed. e.g. fewer URTIs, lower back pain, depression, asthma; more uncomplicated hypertension, dermatitis, weakness/tiredness general, sleep disturbance. Referral by GPs – decreased to physio and exercise therapy, increased to mental health. BUT change is small and limited change in GPs’ objective workload |  |
| 69 | Ekwo, EE  1980  Observation and modelling | To determine the extent to which certain variables explain the variability in the amount of time a PA spends in an office visit/ To define those factors that ought to be taken into account when attempting to establish output categories | All 15 family physicians in the state of Iowa, USA employing 19 PAs.  Informal algorithms for patient management by PA and for consultations with the medical doctor. PAs see walk-in, non urgent and urgent scheduled appointments. 57% of cases with self-limiting, vague or no symptoms. | 1036 patient visits (with times available for 1018). | **Activity level/throughput**  Paper presents detailed descriptive data of the time spent on different clinical presentations and tasks (not presented here).  Actual time spent with PAs– model accounting for 9.2% of variability found age of patient, reason for patient visit, expected source of payment, and number of tasks performed (data collecting, nursing, diagnostic and patient counselling) , observer’s visits, and patient’s payment source to be predictive factors.  **Support and supervision**  PA consulted MD for 11.9% patients (no stat difference at same or satellite sites). | Well described study with detail of method and PA /practice inclusion, strengthened by control for patient and system variables in the statistical analyses.  Authors acknowledge limited in conclusions by the low percentage of variation predicted by the variables, questioning whether variables describing tasks can account for the analytical functions of primary care physicians. |
| 70 | Henry, RA  1972  Survey | To evaluate the impact of PAs in the delivery of primary health care in a physician less community | 1^st^ survey- Female head of households in Gilchrist county; 2^nd^ survey- Patient/parent of the patient  Clinic in Trenton, Gilchrist county, Florida, USA. Staff- 2 PAs (Full-time) with alternated on night and weekend emergency calls; 1 receptionist-bookkeeper; 1 licensed practical nurse; physician supervision (part-time). Clinic served- residents of Gilchrist county (3,500 people) and emergency conditions | 1^st^ survey- 27% of the total county population (not further specified); 2^nd^ survey- Sample size not stated | **Support and supervision** During the 1st 6 months of clinic operation, PAs required consultation for 10% of patients (either with the attending physician or the patient's physician); another 10% of patient cases were discussed by the PAs and the physician; 80% of the patient's visits were handled by the PAs. The PAs used the telephone sparingly to call the attending physicians, although they were always available. In several instances, problems arose beyond the expected competence of the PAs, they referred the patient to a physician |  |
| 71 | Wright  1977  Administrative (cost) data | How is the relationship between quality and cost influenced by medical training? | Patients coming to the clinic with acute illnesses during a 9-month study period  Two model practices run by the Family Practice Residency programme at University of Utah, USA. Providers consisted of 12 first year, 14 second year and 11 third year family practice residents, nine attending physicians on the family practice faculty and two physician assistants | 1700 episodes of acute illness treated in ambulatory care clinics | **Cost**  Average total cost per episode was unrelated to type of provider, but there were significant (*p*<0.05) differences among providers in laboratory (PAs second highest) and medication costs (PAs highest).  PAs achieved more good outcomes (87%, 125 out of 143 cases) than either the faculty or any of the residents, although they saw patients of the same type and degree of severity.  Faculty and PAs had significantly (p<0.05) higher total and laboratory costs for patients with bad outcomes than did any of the residents for patients with good outcomes. Patients with bad outcomes who were treated by PAs had the highest office costs, significantly (*p*<0.05) above any other group of patients with either outcome | Clearly described methods in a large study.  Cost analysed against patient outcomes.  Limited by outcomes based on patient self report, and leaning towards acute conditions in the data, not being representative of family practice overall. |
| 72 | Kane, RL  1978  Structured interviews | To compare the outcomes achieved in a series of acute care episodes by different levels of family practice providers working in the clinic setting | Each patient visiting the centres with any acute complaint in nine months (Oct 1974 to May 1975) to 12 first year, 14 second year, 11 third year family practice residents, nine attending physicians on the family practice faculty and two PAs.  Two family practice centres associated with a university family practice residency programme, USA. | 1761 patient episodes (n=146 by PAs) | **Cost**  Medex most expensive average cost per episode of care (non significant). Had highest mean cost for medication (p=0.004) and second highest (after faculty members) for laboratory costs (p=0.04) | Clear description of methods and tools used.  Limited by cross sectional analysis of each provider group, small number of cases for subset analysis, self reported patient functional status, and being carried out in a small number of practices in a training setting |

**Studies of PROCESS: Patient consultation types, activity levels/throughput, impact on workload of others, support and supervision and cost**
