# Supplementary material for: The contribution of Physician Assistants in primary care: a systematic review
Source: BMC Health Serv Res. 2013 Jun 18;13:223. doi: 10.1186/1472-6963-13-223 (PMC3698179; doi:10.1186/1472-6963-13-223)
Supplement: Additional file 3 — Studies of outcome – data extraction. [file 1472-6963-13-223-S3.docx]

**Additional file 3**

| **Reference number** | **First Author**  **Year**  **Study Design** | **Aim** | **Study population**  **Setting (including country)** | **Sample** | **Key findings** | **Major strengths and limitations** |
| --- | --- | --- | --- | --- | --- | --- |
| 44 | Duttera, MJ  1978  Observation | To describe the practice activities of physician assistants and their physician mentors and to describe their working inter relationships | Physician Assistant graduates from a two tear programme working continuously in rural primary care practice for more than six months in 14 practice sites in six south eastern states, USA | 788 outpatient-provider encounters | **Appropriateness**  Diagnostic and therapeutic appropriateness scores were highly correlated (r=0.87, p<0.001). Scores of PAs were correlated with those of their physician employers (r=0.61, p<0.01). PAs and physicians handled less severe (scores 1 to 5) and more severe (scores 7 to 9) encounters equally well, with no statistically significant difference in performance.  Except for the PAs acting most independently in some practices, there was no apparent trend for independence to be associated with poor performance by PAs (p<0.10). | Selection of these 17 sites (from national survey) not specified.  Observational study may not be large enough to capture all variation. |
| 45 | Henry, LR  2007  Qualitative interviews and focus group | To determine the factors that influence autonomous rural PAs who work < 8 hrs per week with their supervising physician) to remain in remote locations | Community residents of Texas rural towns, USA.  PAs identified through snowball sampling via PA organisations, educational establishments.  PA working autonomously < 8 hours contact with supervising MD. PA sole primary care practitioner in community. PA worked > 24 months in community. Town < 4,000 residents. No other primary health care options within 25 mile radius | 8 towns, 8 PAs, 8 focus groups | **Acceptability to patients**  PAs thought that residents didn’t mind that PA rather than MD but some residents wanted doctor but realised that MD better than nothing. Non patients because 1) PA not doctor, not confident in PA, 2) already had a doctor, 3) had long term conditions which needed specialist care. PAs thought that residents didn’t mind that PA rather than MD but some residents wanted doctor but realised that MD better than nothing. Non patients because 1) PA not doctor, not confident in PA, 2) already had a doctor, 3) had long term conditions which needed specialist care | Well described study.  Limited to one USA state, with potential self-selection bias of participating practices |
| 48 | Ford, VH  1998  Qualitative interview | To examine the perception of family physicians towards nurse practitioners and physician assistants. | Family practice 3 year residency program in Southeast USA linked with a medical school – faculty and resident staff  South East USA | 10 interview: five faculty and five residents | **Appropriateness**  PA education described as medical model while NP nursing model. Some ‘lack of trust’ in the education and ability of NPs, not expressed for PAs. But generally thought to provide high-quality care. Both NP and PA described as taking more time and being more thorough in obtaining patient histories than physicians | Interview (topic) guide is not described.  No respondent transcript excerpts are used to support thematic analysis.  Limited to one university where authors acknowledge NPs had not been trained but PAs were active contributors. |
| 59 | Frame, PS  1978  Medical record review and annual costs | To test whether outcome of diseases treated by the PA is equal to that of the supervising physician | Population - Charts of all patients at the satellite clinic for whom a diagnosis of hypertension was made July 1974 to July 1975 and who made a return visit to the practice  Tri-County Family Medicine (non profit) providing primary care to rural area of western New York State, USA with medical staff of four FPs and one PA. A central practice and 4 satellites to which PA and physicians commute. PA graduate of 2 year New York programme. Receptionist decides if patient condition is in PA job description and offers choice to see PA (sooner than GP). PA in practice for 4 years. | 92 | **Appropriateness**  Three groups – MD at greater than 75% visits (n=44), PA at greater than 75% visits (n=18), MD and PA with no predominance (n=30)  MD group had greater number additional chronic diseases  Overall outcome of hypertensive treatment is not significantly different between groups; all had good control | Limited description of method and samples.  Limited to one practice setting, with restrictions on generalisability.  . |
| 60 | Hill, RF  1979  Medical record review; observation, interviews | To probe parameters of feasibility of the PA role - utilisation, acceptance, quality of care and economics | Patients attending these clinics, PAs and other clinic staff.  Single PA-manned remote clinic (population 1239) in north central Oklahama, USA and three other private rural Oklahama clinics where PAs performed primary care services but the physician was not remote | Approximately  1,000 patient encounters randomly sampled at each site  over a 5-month period | **Acceptability to patients**  96% of patient respondents in Yale clinic were satisfied with the services at the end of the 1^st^ year and nearly a third of this group identified the PA as their "family doctor". Most important reason for using this clinic: 62.7% cited convenience, rather than professional qualities.  **Appropriateness**  Quality of care by remote PA: Low frequency of documentation of quality criteria especially in the "history and physical examination" area | Small study of one PA in one practice.  Methods and sample poorly described |
| 65 | Mainous, AG  1992  Survey – secondary data analysis | To determine the extent of physician extender use and to explore patient factors associated with such use of physician extenders in primary care. | Adults (age 18 or older) living in Kentucky, USA  Random digit telephone dialling and interviewing | Response rate- 68.4%. Usable data-687 individuals | **Acceptability to patients**  20% of respondents used PAs for medical advice or treatment within the past 2 years (most widely used among providers with formal healthcare training). 92% of those using PAs in the preceding 2 years reported being either “somewhat satisfied” or “very satisfied” with the treatment or advice rendered. | Good population coverage with random selection.  Self report – unknown if respondents knew if seen a PA or NP  Limited to one state in USA |
| 72 | Kane, RL  1978  Structured interviews | To compare the outcomes achieved in a series of acute care episodes by different levels of family practice providers working in the clinic setting | Each patient visiting the centres with any acute complaint in nine months (Oct 1974 to May 1975) to 12 first year, 14 second year, 11 third year family practice residents, nine attending physicians on the family practice faculty and two PAs.  Two family practice centres associated with a university family practice residency programme, USA. | 1761 patient episodes (n=146 by PAs) | **Acceptability to patients**  98% seen by PA satisfied with care and 95% with outcome (at point in time physician estimated for full benefit of the treatment to be realised). No significant differences with satisfaction with other provider levels.  **Appropriateness**  Good functional outcomes reported by PA-attended patients for the following conditions:  Headache 100% (n=1)  Otitis 100% (n=10)  Pharyngitis 77% (N=13)  URTI 92% (n=48)  Flu 75% (n=4)  Abdominal pain 60% (n=5)  Rash 67% (n=3)  Neck/back pain 50% (n=2)  Cough 50% (n=2).  No statistically significant differences to other providers | Clear description of methods and tools used.  Limited by cross sectional analysis of each provider group, small number of cases for subset analysis, self reported patient functional status, and being carried out in a small number of practices in a training setting. |
| 73 | Henry, RA  1974  Survey | To evaluate the impact of 2 PAs on the population in the community | Random sample-of 1,700 female heads of household  Clinic in Trenton, Gilchrist county, Florida, USA. Clinic served- residents of Gilchrist county (3,500 people i.e., approx. 800 families) and emergency conditions | 260 clinic users participated in the 2nd survey | **Acceptability of patients**  Provider: Care provided by PA alone- 204 (78%)  Patient acceptance:  1) Liked PAs Very much- 204 (78%), Somewhat- 46 (18%), Not at all- 10 (4%);  2) Ease of talking to PAs as compared to physicians Easier- 80 (31%), Same- 155 (59%), More difficult- 25 (10%)  **Appropriateness**  Analysis of events- Patients were adequately and appropriately treated by the PAs with no required or requested input by a physician in 80% of the visits. | Adequately described methods and results.  Apparent low response rate (difficult to calculate from information)  Appropriateness judgment based on patient self report  Limited to one USA county |
| 74 | Hooker, RS  2005  Survey | Are patients as satisfied with PA and NP care as they are with physician care? | Medicare recipients enrolled in fee-for-service programme for 6 months or longer who identified a generalist physician, PA or NP as their personal health provider on the 2000 or 2001 Medicare consumer survey, aged 65 and over, USA | 3770 respondents who had seen a PA or NP | **Acceptability to patients**  Four satisfaction questions, response range 1-5, poor-excellent:  Response mean 3.5-3.7 physicians, 3.6 to 3.7 PAs, 3.5 to 3.6 NPs. Only 1% of variance accounted for by the provider type. Patients seen by NP or PA less likely to have supplemental insurance than those seen by physicians. | Large national survey with large subsample seen a PA or NP.  Limited to Medicare recipients |
| 75 | Cipher, DJ  2006  Survey – secondary data analysis | To expand on the empirical evidence regarding older patients' experiences with PAs and NPs | Random sample (321,407) of people aged 65 years and older:  Medicare recipients from the 2000 and 2001 Medicare Satisfaction Survey, Consumer Assessment of Health Plans Survey (CAHPS) section on Fee-for-Service (FFS), who identified a primary care provider as a PA, an NP or a generalist physician | Response rate- 45.7% (Completed returns: 146,880)  3770 who identified a PA or an NP as their personal provider used in this analysis | **Acceptability to patients**  Among all beneficiaries,  Health ratings (in fair or poor health) from those recipients who saw an NP (38.7%) were significantly worse (p<0.001) than those from respondents who saw a physician (30.5%) or PA (33.3%).  When recipients were asked to rate their provider, the ratings were consistent across those who received care from a physician, PA, or NP (p=0.03).  The distribution of responses to satisfaction questions was similar across all 3 provider groups. The response means for the 6 satisfaction-related items ranged from 3.5 to 3.7 for physicians, 3.6 to 3.7 for PAs, and 3.5 to 3.6 for NPs. The standard deviation was between 0.55 and 0.70 and the 95% confidence interval was between 3.50 and 3.70 for all 4 questions and all 3 providers. | This study repeats the data presented in Hooker et al, 2005 |
| 76 | Litman TJ  1972  Interview | To assess the potential willingness of a random sample of rural residents to permit specially trained former medical corpsmen to provide various medical services to themselves and their families under the supervision of a physician. | Households served by the community hospitals in Austin and Albert Lea, Minnesota which serves a population of about 115,000 persons, USA | 253 interviews | **Acceptability to patients**  65.6% were willing for PA to take care of themselves or family, 16.4% said they would not. Support for PA based on 1) being adequately trained (17.4%), 2) recognition of heavy demand on local physicians (14.4%) 3) only under doctor’s supervision (12%) 4) confidence in anyone who works with an MD (9.6%) 5) only if MD said so (9.6%). Less agreement over services PA should perform, 94.4% in favour of routine physical examinations and history taking, 76.6% in favour of simple emergency care including sewing up cuts, 58.8% in favour of providing post natal care, 26.4% against pre-natal care, 71% against performing deliveries, 34% against PA screening patient as to whether should be seen by doctor, 83.2% in favour of PA referring | Methods and sample poorly described (e.g. what is the probability sample?)  Unclear if the respondents had previously been treated by PAs or were naive to the concept.  Limited to one geographical area. |
| 77 | Smith, CW  1981  Survey | To examine patients' attitudes toward physicians' assistants and the extent to which patients understand the role and training of the PA | Two hundred patients (50 consecutive in four family practice teams) January 1979.  Multi speciality clinic in south eastern Iowa, USA in a town of 25000 people. Four FPs, each with a PA, one internist and one paediatrician. Tasks delegated to the PA by their supervising physician so varied within the group | 196 completed surveys | **Acceptability to patients**  Overall satisfaction with PAs: 78% satisfied with the team approach physician, PA, nurse; 8% always wanted to see the physician; 26% felt PA could always handle simple problems; 64% felt PA adequately supervised, 62% felt physician was always available for supervision; 26% felt PA helped physician do a better job, 12% to spend more time with patients; 29% at least occasionally uncomfortable with PA.  Satisfaction with different levels of PA care: Varied with complexity of the issue - % patients usually/always comfortable with PA function: consult with physician regarding any problems 87, provide health information 84, treat cold/flu/minor problems 78, help with weight/smoking/etc. 76, answer night calls 73, routine examination 69, instruct re-management of problems 68, order lab test/xray 67, refer to specialist 66, treat in ER 66, handle all patient’s care with physician supervision 55, prescribe 51, chronic conditions 50, family problems 48, diagnose any health problems 48, treat major acute problem e.g. MI 32%. | Adequately described study with high response rate.  Biased towards those who understand PAs through only surveying those attending the clinic.  Limited to one town. |
| 77 continued | Smith continued |  |  |  | Perceptions of the role of PAs: treat minor lacerations 83%,routine physical examinations 80%, diagnose and treat uncomplicated problems 70%, take medical history 70%, take night call 68%, train to become physician62%, extend physician’s care by treating any patient 56%, well-baby examinations 53%, care for patients as nurse 48%.  Perceptions of the training of PAs:% though PA trained to perform – work under physician supervision 76%, physical examination 74%, health information 71%, uncomplicated problems 70%, suture 51%, diagnose medical problems 45%, counsel families 44%, apply casts 40%, prescribe 37%, assist in surgery 26%, deliver babies 11%, practice independently (8%). |  |
| 78 | Hooker, RS  2010  Survey | To test the willingness of patients to be treated by a PA. Are Australians willing to be treated by a PA over a doctor under certain circumstances? | North Queensland, Australia (area where PAs currently unknown). Uni Health Medical Centre, community health clinic, Townsville, Queensland. Clinic operating since 2002, 8 GPs, manages ~ 30,000 patients per year | 225 women, aged 20-50, naive about PAs | **Acceptability to patients**  99% selected to be treated by PA regardless of the scenario. When time differences between doctor and PA were reduced to 2 hours and 1 hour, still preferred PA. Qualitative comments indicate that for long standing condition would prefer GP while injury scenario was different | Well described methods and sample, powered to detect differences.  Hypothetical decision making, with only women of child bearing age included, and use of patient scenarios (e.g. trauma) that may not be applicable to general practice.  Limited to one clinic. |
| 79 | Ohman-Strickland  2008  Medical record review – secondary data analysis | To assess whether the quality of diabetes care differs among practices employing NPs, PAs or neither and which practice attributes contribute to any differences in care. | 20 patient’s charts chosen randomly from each practice of all adults treated with type I or type II diabetes in past year. 732 staff members.  Family medical practices in New Jersey and Pennsylvania, USA | 26 practices, 17 ith a PA and 9 with a NP. 846 patients with diabetes. 452 staff (62% RR) | **Appropriateness**  Compared to practice with PAs, NPs more likely to measure haem (66% vs 33%), lipid levels (80% vs 58%) urinary microalbumin levels (32% vs 6%), treated high lipid levels (77% vs 56%0, patients attain lipid targets (54% vs 37%). Practices with PAs or NPs busier and larger than physician only. Compare PA and physician only. PA 67% less likely to assess microalbim levels, 32% and 15% less likely to assess HbA and lipid levels, patient 32% less likely to attain lipid targets | Well described study, controlled for confounding factors in analysis.  Authors report relatively small numbers of practices may have limited ability to observe differences, and observational study not able to establish causation |

**Studies of OUTCOME: Acceptability to patients, appropriateness of care and cost effectiveness**
